# PReS-FINAL-2260: Provisional findings of an on-going study of musculoskeletal anomalies in a national cohort of patients with trisomy 21

**DOI:** 10.1186/1546-0096-11-S2-P250

**Published:** 2013-12-05

**Authors:** C Foley, OG Killeen

**Affiliations:** 1 OLCHC/NCRC, Dublin, Ireland; 2 OLCHC, Dublin, Ireland

## Introduction

Musculoskeletal complications of Down syndrome are common. Joint laxity is almost universal. This, in combination with low muscle tone, contribute to increased risk of a number of musculoskeletal disorders e.g. atlanto-axial instability, patella instability & pes planus. Arthritis in children with Down syndrome is also reported. Down's Arthropathy (DA) is thought to be 3-6 times more common than JIA in the general paediatric population. Despite this fact, DA is rarely recognised at onset & remains under-diagnosed. This contributes to unnecessary disability & functional impairment.

## Objectives

1.Take a musculoskeletal history from, and perform a musculoskeletal examination on children with Down syndrome between the ages of 0.5-18 years. 2.Score hypermobility using the Beighton & Brighton screening tools. 3.Examine joints for evidence of past and/or present arthritis.

## Methods

From April 2013 to April 2014, children with Down syndrome will be invited to attend a screening clinic. Screening involves completion of a health questionnaire & a comprehensive musculoskeletal exam.

## Results

Our results support a feature consistently reported in the limited literature available on DA (table 1). There is delayed diagnosis, leading to less favourable outcomes. The average time to diagnosis in our cohort was 1.9 years, with the longest delay reported nearly 5 years. This child developed loss of joint space, generalized osteopenia, erosions & subluxations of affected joints. When compared with a cohort of our newly diagnosed JIA patients (time to diagnosis 0.6 years), we demonstrate a significant difference in time to diagnosis (p = 0.025). To date 59 children have enrolled in the musculoskeletal screening process, 69% of whom have pes planus. No atlanto-axial instability has been reported, however one child had an absent C2 vertebra. There has been one case of patella instability and 4 new cases of DA diagnosed. Anecdotally we have not found the Beighton & Brighton criteria comprehensive. The majority of children were found to have hypermobile hips. Neither scoring systems incorporate hips in their screening criteria. To date 100% of children screened would not have BHS using Brighton criteria.

**Table 1:** 

		Average	**Std. Dev**.	Minimum	Maximum
**Current Down's Arthropathy Cohort** n = 12	**Age **(years)	8.5	4.5	0.3	14.9
	Active Joint Count*	6	3.7	1	11
	Restricted Joint Count*	3	3.3	0	12
	**Delay in Diagnosis **(years)	1.9	1.5	0.2	4.9 *At Presentation

## Conclusion

Pes planus is commonly seen in children with T21, therefore orthotics & advice regarding correct footwear is important. Children with T21 often have hypermobile hips, not accounted for by the current scoring criteria for BHS. DA is common but often missed, with delayed diagnosis. Early diagnosis & treatment of DA is important to prevent unwanted joint destruction & functional disability. Children with T21 should have a musculoskeletal exam as part of their annual screening programme.

## Disclosure of interest

None declared.

